# Phloroglucinol Attenuates DNA Damage and Apoptosis Induced by Oxidative Stress in Human Retinal Pigment Epithelium ARPE-19 Cells by Blocking the Production of Mitochondrial ROS

**DOI:** 10.3390/antiox11122353

**Published:** 2022-11-28

**Authors:** Cheol Park, Hee-Jae Cha, Min Yeong Kim, EunJin Bang, Sung-Kwon Moon, Seok Joong Yun, Wun-Jae Kim, Jeong Sook Noh, Gi-Young Kim, Suengmok Cho, Hyesook Lee, Yung Hyun Choi

**Affiliations:** 1 Division of Basic Sciences, College of Liberal Studies, Dong-Eui University, Busan 47340, Republic of Korea; 2 Department of Parasitology and Genetics, Kosin University College of Medicine, Busan 49267, Republic of Korea; 3 Anti-Aging Research Center, Dong-Eui University, Busan 47340, Republic of Korea; 4 Department of Biochemistry, College of Korean Medicine, Dong-Eui University, Busan 47227, Republic of Korea; 5 Department of Food and Nutrition, Chung-Ang University, Ansung 17546, Republic of Korea; 6 Department of Urology, College of Medicine, Chungbuk National University, Cheongju 28644, Republic of Korea; 7 Department of Food Science & Nutrition, Tongmyong University, Busan 48520, Republic of Korea; 8 Department of Marine Life Science, Jeju National University, Jeju 63243, Republic of Korea; 9 Department of Food Science and Technology, Institute of Food Science, Pukyong National University, Busan 48513, Republic of Korea; 10 Department of Convergence Medicine, Pusan National University School of Medicine, Yangsan 50612, Republic of Korea

**Keywords:** phloroglucinol, mitochondrial ROS, DNA damage, autophagy, apoptosis

## Abstract

Phloroglucinol, a phenolic compound, is known to possess a potent antioxidant ability. However, its role in retinal cells susceptible to oxidative stress has not been well elucidated yet. Thus, the objective of this study was to evaluate whether phloroglucinol could protect against oxidative damage in cultured human retinal pigment epithelium ARPE-19 cells. For this purpose, ARPE-19 cells were stimula ted with hydrogen peroxide (H_2_O_2_) to mimic oxidative stress. Cell viability, cytotoxicity, apoptosis, reactive oxygen species (ROS) generation, mitochondrial function, DNA damage, and autophagy were then assessed. Our results revealed that phloroglucinol ameliorated cell viability, cytotoxicity, and DNA damage in H_2_O_2_-exposued ARPE-19 cells and blocked production of ROS. Phloroglucinol also counteracted H_2_O_2_-induced apoptosis by reducing Bax/Bcl-2 ratio, blocking activation of caspase-3, and inhibiting degradation of poly (ADP-ribose) polymerase. H_2_O_2_ caused mitochondrial impairment and increased expression levels of mitophagy markers such as PINK1and PARKIN known to be associated with mitochondrial ROS (mtROS) generation and cytosolic release of cytochrome *c*. However, these changes were significantly attenuated by phloroglucinol. Mito-TEMPO, a selective mitochondrial antioxidant, further enhanced the protective effect of phloroglucinol against dysfunctional mitochondria. Furthermore, H_2_O_2_ induced autophagy, but not when ARPE-19 cells were pretreated with phloroglucinol, meaning that autophagy by H_2_O_2_ contributed to the pro-survival mechanism and that phloroglucinol protected ARPE-19 cells from apoptosis by blocking autophagy. Taken together, these results suggest that phloroglucinol can inhibit oxidative stress-induced ARPE-19 cell damage and dysfunction by protecting DNA damage, autophagy, and subsequent apoptosis through mitigation of mtROS generation. Thus, phloroglucinol might have therapeutic potential to prevent oxidative stress-mediated damage in RPE cells.

## 1. Introduction

The retina expends excessive energy for the formation of visual perception. It is very sensitive to oxidative stress. At the same time, the retina serves as a powerful generator of reactive oxygen species (ROS) implicated in several major retinal diseases, including age-related macular degeneration (AMD), a leading cause of vision loss [1,2]. Although the etiology and mechanism of AMD induction remain unclear, oxidative stress-related injury to the retinal pigment epithelium (RPE) is recognized as an early event in in AMD-like pathology [3,4]. Appropriate levels of intracellular ROS including mitochondrial ROS (mtROS) play important physiological roles as modulators of cellular signaling pathways. However, excessive accumulation of ROS by persistent oxidative stress can lead to cellular injury and death and contribute to the initiation of pathological damage to various organs, including eyes [5,6]. In addition, apoptosis and autophagy of RPE cells due to excessive ROS production are accompanied by mitochondrial and DNA damage, ultimately contributing to retina dysfunction [7,8]. Furthermore, since mitochondrial damage in RPE degeneration can induce a cellular defense mechanism known as mitophagy, mitophagy could be a putative therapeutic target in retinal degenerative diseases such as AMD [9,10]. Therefore, the level of ROS must be tightly controlled to protect normal functions of eyes.

Natural resources have long received great attention as sources of drug development. Among them, phenolic compounds derived from natural products having excellent antioxidant activity have attracted attention. Their antioxidant activities mainly involve scavenging of ROS and activation of intracellular antioxidant signaling pathways [5,11,12]. Phloroglucinol, a polyphenol trihydroxybenzene with an aromatic phenyl ring and three hydroxyl groups, is a naturally occurring secondary metabolite present in a variety of organisms including plants, algae, and bacteria [13,14]. This phenolic compound is known to have various pharmacological potentials such as antibacterial, anticonvulsant, anti-allergic, antithrombotic, anti-inflammatory, and cancer chemopreventive activities [15,16]. Recently, the antioxidant potential of phloroglucinol has been validated in several in vitro and in vivo models. For example, Drygalski et al. [17] have reported that phloroglucinol can strengthen antioxidant defense and ameliorate hepatic steatosis and inflammatory response by reducing oxidative/nitrogen damage to cellular macromolecules. In addition, it has been confirmed that phloroglucinol can block oxidative damage caused by hydrogen peroxide (H_2_O_2_) treatment and γ-ray irradiation by regulating activities of antioxidant and detoxifying enzymes in the retinal epithelium, hippocampal nerve, renal epithelial cells, and lung fibroblasts [18,19,20]. Moreover, phloroglucinol as an ROS scavenger can modulate synaptic plasticity to attenuate pathological phenomena of neurodegenerative diseases such as Alzheimer’s disease and Parkinson’s disease [21,22]. Our previous study has shown that phloroglucinol can inhibit DNA damage and apoptosis in H_2_O_2_-exposed HaCaT human keratinocytes [23]. Similar results have been confirmed in ultraviolet (UV) B-irradiated keratinocytes and all-trans-retinal-exposed primary rat RPE and mouse photoreceptor cells [19,24]. Recently, Kuo et al. [25] have reported that phloroglucinol can block oxidative cytotoxicity induced by potassium bromate, an AMD inducer, in human RPE ARPE-19 cells by inhibiting ROS production. These results suggest that phloroglucinol could play an antioxidant role in ARPE-19 cells as suggested by Moine et al. [26]. Nevertheless, studies on the protective role of phloroglucinol against cellular damage induced by oxidative stress in RPE cells are lacking. Therefore, the purpose of the current study was to investigate effects of phloroglucinol on oxidative stress-induced mitochondrial and DNA damage and induction of apoptosis and autophagy in RPE cells. For this purpose, a human RPE-derived ARPE-19 cell model treated with H_2_O_2_ to mimic oxidative stress was used.

## 2. Materials and Methods

### 2.1. Cell Culture and Treatment

ARPE-19 cells (CRL-2302) were purchased from the American Type Culture Collection (Manassas, VA, USA) and routinely cultured in Dulbecco’s Modified Eagle Medium/F-12 supplemented with 10% fetal bovine serum and 1% penicillin-streptomycin (WELGENE Inc., Gyeongsan, Republic of Korea) as described previously [27]. To investigate beneficial effects of phloroglucinol on oxidative damage, cells were cultured in media containing desired concentrations of phloroglucinol and H_2_O_2_ (Thermo Fisher Scientific, Inc., Waltham, MA, USA) for 24 h or pretreated with phloroglucinol, N-acetyl-L-cysteine (NAC), Mito-TEMPO, and/or 3-methyladenine (3-MA, Sigma-Aldrich Co., St. Louis, MO, USA) for 1 h prior to treatment with H_2_O_2_ for 24 h. To investigate the blocking effect of phloroglucinol on the generation of ROS induced by H_2_O_2_, cells were pretreated with phloroglucinol, NAC, and Mito-TEMPO for 1 h and then treated with H_2_O_2_ for 1 h. To acquire fluorescence images of ROS generation, γH2AX expression, and autophagic vacuoles, cells cultured on coverslips were stimulated with H_2_O_2_ in the presence or absence of phloroglucinol, NAC, and/or Mito-TEMPO. After treatment, cells were washed with phosphate-buffered saline and subjected to fluorescence staining.

### 2.2. Cell Viability Assay

To investigate viability of ARPE-19 cells cultured under various conditions, 3-(4,5-dimethylthiazol-2-yl)-2,5-diphenyltetra-zolium bromide (MTT) assay was performed. In brief, after the necessary experimental treatment, cells were incubated with MTT solution (Thermo Fisher Scientific, Inc.) for 3 h. Formed insoluble formazan products were then dissolved in dimethyl sulfoxide (DMSO, Thermo Fisher Scientific, Inc.) and the absorbance was read at 570 nm using an enzyme-linked immunosorbent assay (ELISA) microplate reader (Molecular Device Co., Sunnyvale, CA, USA) according to a previously described method [28]. Cell viability was expressed as a percentage of untreated control cells.

### 2.3. Cytotoxicity Assay

To assess cytotoxicity, lactate dehydrogenase (LDH) release was detected using an LDH Cytotoxicity Assay Kit (Thermo Fisher Scientific, Inc.) according to the manufacturer’s instructions. In brief, culture medium obtained from conditions treated with H_2_O_2_ in the presence or absence of phloroglucinol was transferred to a 96-well plate and the amount of released LDH was measured at 490 nm with an ELISA microplate reader.

### 2.4. Quantitative Assessment of Apoptosis

Annexin V-Fluorescein Isothiocyanate (FITC) Apoptosis Detection Kit was purchased from Abcam Inc. (Cambridge, UK) and used for quantitative evaluation of apoptosis-induced cells upon treatment with phloroglucinol and/or H_2_O_2_. After treatment, collected cells were suspended in annexin binding buffer containing annexin V- FITC and propidium Iodide (PI) following the manufacturer’s instructions. The fluorescence of 10,000 events was then acquired using a flow cytometer (Becton Dickinson, San Jose, CA, USA). Annexin V-positive cells were considered as apoptosis-induced cells as described previously [27].

### 2.5. DNA Fragmentation Assay

To observe fragmented DNA, an apoptosis marker, cell pellet was suspended in a lysis solution as described previously [29]. The supernatant was then incubated with RNase A and proteinase K (Sigma-Aldrich Co.). DNA was precipitated with isopropyl alcohol (Sigma-Aldrich Co.). The extracted DNA was fractionated using 1.0% agarose gel and then stained with ethidium bromide (EtBr, Thermo Fisher Scientific, Inc.) to visualize DNA fragmentation pattern, a characteristic of apoptosis, under UV light.

### 2.6. Protein Isolation and Western Blotting

Total protein to be used for Western blot analysis was extracted as described previously [30]. Cytoplasmic and mitochondrial proteins were isolated using a Mitochondrial Fractionation Kit (Thermo Fisher Scientific, Inc.) following the manufacturer’s instructions. In brief, proteins were separated by sodium dodecyl sulfate-polyacrylamide gel electrophoresis and transferred to Immobilon^®^-P PVDF membranes (Merck Millipore, Bedford, MA, USA). These membranes were then incubated with specific primary antibodies overnight followed by reaction with horseradish peroxidase-conjugated secondary antibodies (Santa Cruz Biotechnology, Inc., Santa Cruz, CA, USA) at room temperature for 1 h. Immune complexes were visualized with enhanced chemiluminescence reagent (Thermo Fisher Scientific, Inc.) according to the manufacturer’s instruction [31]. Densitometric analysis of the data was performed using the ImageJ^®^ software (v1.48, NIH, Bethesda, MD, USA). Primary antibodies were obtained from Santa Cruz Biotechnology, Inc. and Cell Signaling Technology (Beverly, MA, USA). All antibodies used in this study are listed in Table 1.

### 2.7. Caspase-3 Activity Assay

Caspase 3 activity was quantified using a Caspase-3 Colorimetric Assay Kit (Abcam, Inc.). In brief, aliquots of cytosolic extracts were mixed with a fluorescent substrate of caspase-3, acetyl-Asp-Glu-Val-Asp-chromophore-p-nitroanilide (Ac-DVAD-pNa), in the buffer provided in the kit according to the manufacturer’s instructions. Enzyme-catalyzed release of pNa was monitored at 405 nm using an ELISA microplate reader. The activity of caspase-3 was presented relative to the control [32].

### 2.8. Assessment of ROS Generation

Levels of intracellular ROS and mtROS production were detected using fluorescent probes 2′,7′-dichlorofluorescein diacetate (DCF-DA) and MitoSOX (Sigma-Aldrich Co.), respectively. Following exposure to H_2_O_2_ with or without phloroglucinol, NAC, and/or Mito-TEMPO, cells were reacted with DCF-DA and MitoSOX to assess levels of intracellular and mitochondrial peroxides, respectively, using flow cytometry. In parallel, fluorescence images of DCF-DA- and MitoSOX-stained cells cultured on coverslips were observed under a fluorescence microscope (Carl Zeiss, Oberkochen, Germany) at Core-Facility Center for Tissue Regeneration, Dong-eui University (Busan, Republic of Korea).

### 2.9. Comet Assay

The inhibitory effect of phloroglucinol on H_2_O_2_-induced DNA damage after appropriate treatment was determined using comet assay (single cell gel electrophoresis). Briefly, collected cells were suspended in 1% low melting point agarose and then spread on comet slides according the manufacturer’s protocol in of a commercially available Comet Assay Kit (Trevigen, Inc., Gaithersburg, MD, USA). After DNA denaturation, electrophoresis was performed and slides were stained with an asymmetric cyanine dye. Resulting images were acquired under a fluorescence microscope.

### 2.10. γH2AX Immunofluorescence Assay

Immunofluorescence assay was applied to analyze the expression of phosphorylated histone H2AX (p-γH2AX) in cells treated with or without phloroglucinol or NAC before adding H_2_O_2_. Following treatment, cells were fixed with formaldehyde, permeabilized with Triton X-100 solutions (Thermo Fisher Scientific, Inc.), and then blocked with bovine serum albumin solution (Sigma-Aldrich Co.). Thereafter, cells were probed with an anti-p-γH2AX antibody (Cell Signaling Technology, Inc.) and then reacted with Alexa Fluor 555-conjugated secondary antibody (Thermo Fisher Scientific, Inc.). For nuclear counterstaining, cells were immersed in a 4’,6-diamidino-2-phenylindol (DAPI) solution (Sigma-Aldrich Co.). Then, p-γH2AX and DAPI fluorescence images were captured using a fluorescence microscope.

### 2.11. Measurement of 8-Hydroxy-2′-Deoxyguanosine (8-OHdG) 

To measure 8-OHdG, a deoxyriboside form of 8-oxoGuanine, an OxiSelect Oxidative DNA Damage ELISA Kit (Cell Biolabs, San Diego, CA, USA) was used. Briefly, DNA was extracted from cells cultured under the same conditions as described above. Subsequently, the DNA of each isolated sample was digested with DNase I (Sigma-Aldrich Co.). The absorbance of the ELISA reaction was then measured at 450 nm following the protocol presented in the kit.

### 2.12. Mitochondrial Membrane Potential (MMP) Measurement

MMP level was monitored by staining with 5,5′,6,6′-tetrachloro-1,1′3,3′-tetraethyl- imidacarbocyanune iodide (JC-1), a fluorescent carbocyanine probe. For this assay, cells treated with H_2_O_2_ in the presence or absence of phloroglucinol or Mito-TEMPO were stained with JC-1 solution (Thermo Fisher Scientific, Inc.). The percentage of JC-1 monomer was analyzed with a flow cytometer to indicate cells that lost MMP.

### 2.13. Autophagy Detection

Formation of autophagosomes was assessed using a CYTO-ID^®^ Autophagy Detection Kit purchased from Enzo Life Sciences, Inc. (Farmingdale, NY, USA). First, cells were collected for quantitative analysis of autophagy induction. Cyto-ID staining procedure was then performed according to the manufacturer’s instructions. In brief, cells cultured under various conditions were washed with assay buffer included in the kit and fixed with paraformaldehyde. Fluorescently labeled cells were then analyzed by flow cytometry. Next, cells were subjected to DAPI staining after CYTO-ID staining to monitor localizations of autophagosomes and nuclei. To monitor localizations of autophagosomes and nuclei, cells were further subjected to DAPI staining after CYTO-ID staining. The autophagic signal (green) and the nuclear signal (blue) were collected under a fluorescence microscope.

### 2.14. Statistical Analysis

All statistical analyses were performed using GraphPad Prism (Graphpad Inc., San Diego, CA, USA). Statistical differences were determined by one-way analysis of variance with Tukey’s test. Statistical significance was considered when *p*-value was less than 0.05. All data are expressed as mean ± standard deviation (SD) (* *p* < 0.05, ** *p* < 0.01 and *** *p* < 0.001 vs. unstimulated control; ^##^ *p* < 0.01 and ^###^ *p* < 0.001 vs. H_2_O_2_ alone treatment; ^&^ *p* < 0.05 and ^&&&^ *p* < 0.001 vs. phloroglucinol + Mito-TEMPO group).

## 3. Results

### 3.1. Phloroglucinol Restores Reduced Cell Viability and Increased Cytotoxicity Caused by H_2_O_2_

**Figure 1:**
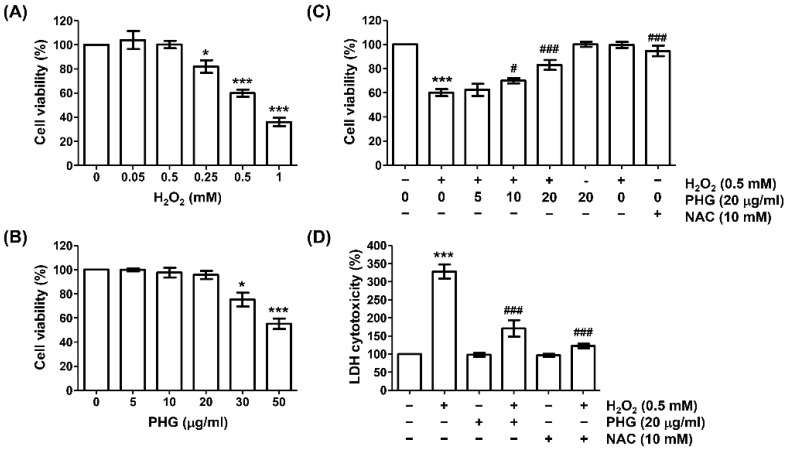
Phloroglucinol reverses H_2_O_2_-induced viability reduction and cytotoxicity of ARPE-19 cells. Cells were treated with different concentrations of phloroglucinol (PHG) or H_2_O_2_ alone for 24 h (**A**,**B**), or pretreated with or without phloroglucinol and/or N-acetyl-L-cysteine (NAC) for 1 h followed by treatment with phloroglucinol for 24 h (**C**,**D**). (**A**–**C**) 3-(4,5-dimethylthiazol-2-yl)-2,5-diphenyltetra-zolium bromide (MTT) assay was performed to determine cell viability. (**D**) Cytotoxicity was measured by lactate dehydrogenase (LDH) assay. * *p* < 0.05 and *** *p* < 0.001 vs. unstimulated control; ^#^ *p* < 0.05 and ^###^ *p* < 0.001 vs. H_2_O_2_ alone treatment.

We performed an MTT assay to select the concentration of H_2_O_2_ that could induce oxidative damage in ARPE-19 cells. As expected, H_2_O_2_ treatment significantly suppressed cell viability in a dose-dependent manner (Figure 1A). At concentration of 0.5 mM, H_2_O_2_ reduced the cell viability to be about 60% of the control group (untreated cells). Thus, 0.5 mM was set as the cytotoxicity-inducing concentration of H_2_O_2_. In addition, in an experiment to determine the appropriate concentration range of phloroglucinol to evaluate its inhibitory effect on H_2_O_2_-induced cytotoxicity, it was found that phloroglucinol had no significant effect on cell survival at concentration up to 20 μg/mL (Figure 1B). Therefore, the highest optimal concentration of phloroglucinol was determined to be 20 μg/mL. Subsequently, we assessed the inhibitory effect of phloroglucinol on H_2_O_2_-induced cytotoxicity and found that phloroglucinol significantly restored H_2_O_2_-induced reduction in cell viability (Figure 1C). In parallel, pretreatment with NAC, an ROS scavenger used as a positive control, also completely inhibited the downregulation of cell viability in response to H_2_O_2_, demonstrating that oxidative stress triggered by H_2_O_2_ might mediate H_2_O_2_-induced reduction in cell viability. Further analysis of the protective effect of phloroglucinol using the LDH leakage assay showed that phloroglucinol and NAC significantly reduced H_2_O_2_-induced LDH release into the cell culture medium (Figure 1D).

### 3.2. Phloroglucinol Reveres H_2_O_2_-Induced Apoptosis

**Figure 2:**
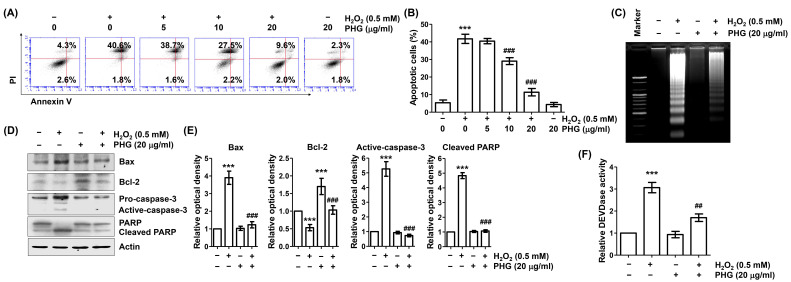
Phloroglucinol inhibits apoptosis in H_2_O_2_-treated ARPE-19 cells. Cells were pretreated with phloroglucinol for 1 h and then treated with or without phloroglucinol for 24 h. (**A**,**B**) To quantitatively measure the frequency of apoptosis induction, flow cytometry was performed after double staining with annexin V and propidium iodide (PI). Representative histograms of (**A**) and quantitative analysis (**B**) are shown. (**C**) DNA isolated from cells was stained with ethidium bromide (EtBr) and then observed under UV light. (**D**) After extracting cell lysate of each treatment group, expression levels of presented proteins were investigated through immunoblotting. (**E**) Bar diagram showing the relative protein density after normalization with actin based on Western blot analysis. (**F**) Activity of caspase-3 was measured by DEVDase activity assay using cytoplasmic extracts and presented as relative values compared to control. *** *p* < 0.001 vs. unstimulated control; ^##^ *p* < 0.01 and ^###^ *p* < 0.001 vs. H_2_O_2_ alone treatment.

We next investigated whether the loss of cell survival and induction of cytotoxicity in H_2_O_2_-exposued ARPE-19 cells were associated with induction of apoptosis. As demonstrated in Figure 2A,B, flow cytometry results after annexin V/PI staining revealed that much more apoptosis was induced in H_2_O_2_-treated cells than in untreated control cells. However, the induction of apoptosis by H_2_O_2_ was remarkably attenuated in cells treated with phloroglucinol. Subsequently, DNA fragmentation assay was performed to verify that phloroglucinol prevented H_2_O_2_-induced apoptosis. As shown in Figure 2C, H_2_O_2_-treated cells exhibited DNA laddering and oligonucleosome-sized DNA fragments. Such patterns were not observed in untreated control cells. However, these patterns were markedly attenuated in cells preincubated with phloroglucinol. H_2_O_2_ treatment also suppressed the Bcl-2/Bax ratio known to be correlated with activation of caspase-3 and cleavage of poly(ADP-ribose) polymerase (PARP), a representative substrate protein of activated caspase-3. However, these changes were greatly ameliorated in cells pretreated with phloroglucinol (Figure 2D–F). These results suggest that phloroglucinol can effectively reduce H_2_O_2_-induced ARPE-19 cell apoptosis by modulating apoptosis regulators.

### 3.3. Phloroglucinol Abrogates H_2_O_2_-Induced ROS Generation

**Figure 3:**
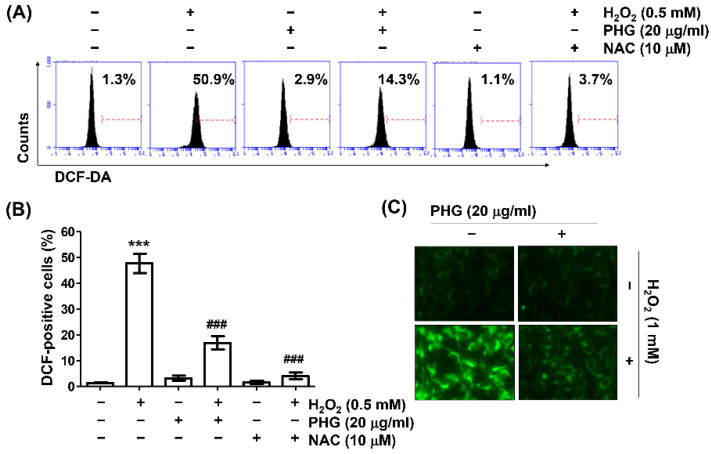
Phloroglucinol suppresses H_2_O_2_-induced reactive oxygen species (ROS) production in ARPE-19 cells. Cells treated with or without phloroglucinol or NAC for 1 h were stimulated with H_2_O_2_ for another 1 h. After 2′,7′-dichlorofluorescein diacetate (DCF-DA) staining, ROS generation level was investigated through flow cytometry (**A**,**B**) and fluorescence microscopy (**C**). Representative histograms (**A**) and quantitative analysis (**B**) are shown. (**C**) Representative images of DCF-DA fluorescence are presented. *** *p* < 0.001 vs. unstimulated control; ^###^ *p* < 0.001 vs. H_2_O_2_ alone treatment.3.4. Phloroglucinol Abolishes H_2_O_2_-Induced DNA Damage.

To clarify antioxidant properties of phloroglucinol, intracellular ROS generation was determined using DCFH-DA probe. As exhibited in Figure 3A,B, results of flow cytometry indicated that the production of intracellular peroxides in cells treated with H_2_O_2_ was greatly increased compared to that in untreated control cells, whereas it was significantly inhibited by pretreatment with phloroglucinol. In addition, ROS hardly occurred in cells treated with phloroglucinol alone. H_2_O_2_ treatment was unable to increase ROS levels in cells treated with phloroglucinol, even in the presence of NAC, indicating that phloroglucinol could act as an ROS scavenger. These results were confirmed through fluorescence microscopic observation of cells stained with DCF-DA. It was found pretreatment with phloroglucinol significantly scavenged H_2_O_2_-induced DCF fluorescence intensity (Figure 3C).

### 3.4. Phloroglucinol Abolishes H_2_O_2_-Induced DNA Damage

**Figure 4:**
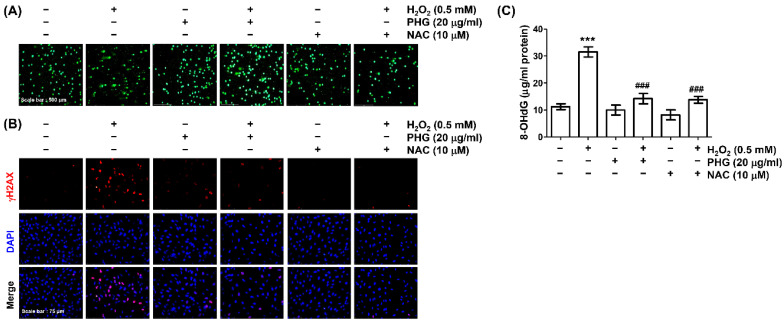
Phloroglucinol alleviates H_2_O_2_-induced DNA damage in ARPE-19 cells. Cells were treated with or without phloroglucinol or NAC for 1 h prior to treatment with H_2_O_2_ for 24 h. (**A**) Representative immunofluorescence images of comet assay are indicated. (**B**) Representative images of γH2AX immunofluorescence (red) observed with a fluorescence microscope are shown. The location of the nucleus was indicated by counterstaining with DAPI (blue). (**C**) After treatment, contents of 8-hydroxy-2′-deoxyguanosine (8-OHdG) were measured using an ELISA kit. *** *p* < 0.001 vs. unstimulated control; ^###^ *p* < 0.001 vs. H_2_O_2_ alone treatment.

Next, we evaluated whether phloroglucinol could prevent H_2_O_2_-induced DNA damage in ARPE-19 cells. The blocking effect of phloroglucinol on DNA damage induced by H_2_O_2_-treatment was first investigated using a comet assay. As expected, an increase in comet tail moment was clearly observed in H_2_O_2_-treated cells, indicating that DNA damage was induced by H_2_O_2_ treatment (Figure 4A). To validate this finding, the expression of γH2AX was analyzed. Immunofluorescence results demonstrated that the fluorescence intensity of γH2AX in the nuclei of H_2_O_2_-treated cells was clearly increased compared to that in the nuclei of untreated cells. Further testing to quantify oxidative damage to nucleic acids also showed that levels of 8-OHdG were significantly elevated upon exposure to H_2_O_2_ (Figure 4C). However, increases of DNA migration, γH2AX expression, and 8-OHdG/8-oxoGuanine ratio caused by H_2_O_2_ treatment were markedly weakened in the presence of NAC as well as phloroglucinol, suggesting that phloroglucinol could attenuated oxidative DNA damage caused by H_2_O_2_.

### 3.5. Phloroglucinol Reduces H_2_O_2_-Induced mtROS Production

**Figure 5:**
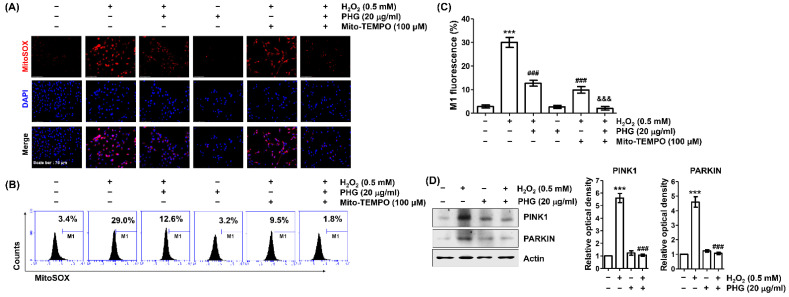
Phloroglucinol eliminates H_2_O_2_-mediated mtROS generation in ARPE-19 cells. MitoSOX staining was performed to determine the abundance of mtROS in cells treated with H_2_O_2_ for 24 h after pretreatment for 1 h with or without phloroglucinol and/or Mito-TEMPO. (**A**) Representative images of cells stained for mitochondrial peroxide (MitoSOX, red) and nuclei (4’,6-diamidino-2-phenylindol, DAPI, blue) are shown. (**B**,**C**) For quantitative evaluation of mtROS production, flow cytometric analysis was performed. Representative histograms (**B**) and average values (**C**) are shown. (**D**) Isolated total cell lysates were immunoblotted with antibodies corresponding to indicated mitophagy-marker proteins. (*** *p* < 0.001 vs. unstimulated control; ^###^ *p* < 0.001 vs. H_2_O_2_ alone treatment; ^&&&^ *p* < 0.001 vs. phloroglucinol + Mito-TEMPO group.

To determine whether mitochondria are major sources of H_2_O_2_-induced ROS and whether phloroglucinol can inhibit them, we used MitoSOX-red, a mitochondrial superoxide-specific dye. As shown in Figure 5A, strong red fluorescence intensity was evident in H_2_O_2_-treated ARPE-19 cells, but not in untreated control cells or cells treated with phloroglucinol alone. However, H_2_O_2_-induced fluorescence intensity was suppressed in the presence of phloroglucinol. It was further eliminated in cells pretreated with phloroglucinol and Mito-TEMPO, a mitochondria-targeted antioxidant. These results were consistent with flow cytometry results, which directly measured the frequency of MitoSOX-red-positive cells (Figure 5B,C). Moreover, phloroglucinol suppressed H_2_O_2_-induced accumulation of PINK1 and PARKIN known to be key mitochondrial autophagy proteins (Figure 5D). These findings suggest that phloroglucinol can contribute to mitophagy inhibition through its role as a scavenger of H_2_O_2_-induced mtROS in ARPE-19 cells.

### 3.6. Phloroglucinol Protects H_2_O_2_-Induced Mitochondrial Impairment

**Figure 6:**
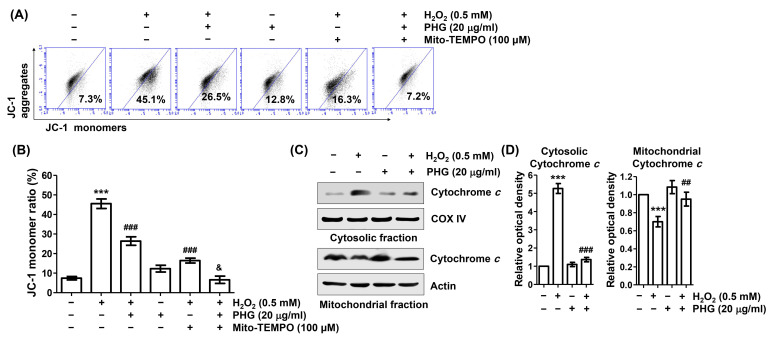
Phloroglucinol protects H_2_O_2_-induced mitochondrial impairment and cytosolic release of cytochrome *c* in ARPE-19 cells. Cells were preincubated with or without phloroglucinol and/or Mito-TEMPO for 1 h, followed by treatment with H_2_O_2_ for another 24 h. (**A**,**B**) After with 5,5′,6,6′-tetrachloro-1,1′3,3′-tetraethyl-imidacarbocyanune iodide (JC-1) staining, representative histograms (**A**) and average values of JC-1 monomer ratios (**B**) are presented. (**C**) After isolation of mitochondrial and cytoplasmic fractions, the expression of cytochrome *c* in each fraction was investigated by immunoblotting. (**D**) Bar diagram showing the relative protein density after normalization with actin based on Western blot analysis. *** *p* < 0.001 vs. unstimulated control; ^##^ *p* < 0.01 and ^###^ *p* < 0.001 vs. H_2_O_2_ alone treatment; ^&^ *p* < 0.05 vs. phloroglucinol + Mito-TEMPO group.

To determine whether phloroglucinol could protect against H_2_O_2_-induced mitochondrial damage, we estimated MMP following JC-1 staining. Flow cytometry analysis results (Figure 6A,B) showed that the frequency of JC-1 monomer was significantly increased whereas the frequency of JC-1 aggregates was decreased in H_2_O_2_-treated cells, indicating that H_2_O_2_ induced the collapse of MMP. However, these changes were significantly attenuated by phloroglucinol pretreatment. Moreover, when phloroglucinol and Mito-TEMPO were used for pretreatment together, the loss of MMP induced by H_2_O_2_ was almost entirely reduced to the control level. In addition, after H_2_O_2_ treatment, the expression level of cytochrome *c* was increased in the cytoplasm but decreased in the mitochondria. Phloroglucinol pretreatment was able to restore these changes (Figure 6C,D). These results illustrate that blockade of H_2_O_2_-induced mtROS generation by phloroglucinol can preserve mitochondrial function.

### 3.7. Phloroglucinol Abrogates H_2_O_2_-Induced Autophagy

**Figure 7:**
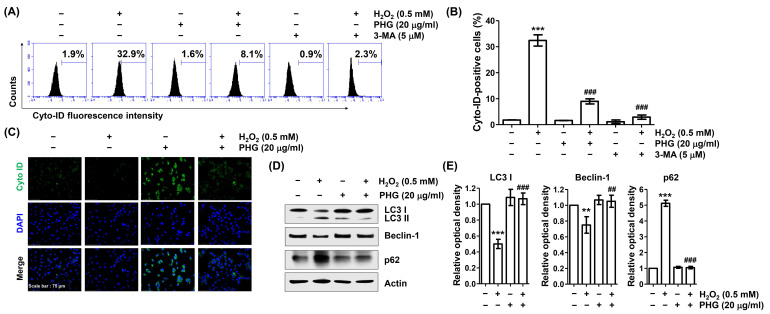
Phloroglucinol attenuates ARPE-19 cells against H_2_O_2_-induced autophagy. (**A**,**B**) Cells were incubated with phloroglucinol or 3-MA for 1 h and then treated with H_2_O_2_ for 24 h, stained with Cyto-ID, and subjected to flow cytometry. Representative histograms (**A**) and mean values of Cyto-ID-positive cells (**B**) are presented. (**C**) H_2_O_2_-teated Cells in the presence or absence of phloroglucinol were stained with Cyto-ID. Representative images are shown. (**D**) Isolated total proteins were immunoblotted with indicated antibodies corresponding to autophagy-marker proteins. (**E**) Bar diagram showing the relative protein density after normalization with actin based on Western blot analysis. ** *p* < 0.01 and *** *p* < 0.001 vs. unstimulated control; ^##^ *p* < 0.01 and ^###^ *p* < 0.001 vs. H_2_O_2_ alone treatment.

Finally, we evaluated the effect of phloroglucinol on H_2_O_2_-induced autophagy in ARPE-19 cells. As shown in Figure 7A,B, flow cytometry analysis using a Cyto-ID tracer dye capable of monitoring autophagic vacuoles showed that H_2_O_2_ dramatically induced autophagy. However, pretreated with phloroglucinol or 3-MA, a selective autophagic inhibitor, dramatically reduced H_2_O_2_-induced autophagy in cells. Consistent with these results, the formation of Cyto-ID puncta was enhanced in response to H_2_O_2_, whereas it was reduced almost completely after phloroglucinol pretreatment (Figure 7C), indicating that H_2_O_2_-induced autophagy could be reversed by phloroglucinol. We next verified H_2_O_2_-induced autophagy by detecting autophagy biomarkers such as microtubule-associated protein-1 light chain-3 (LC3), Beclin-1, and p62 by immunoblotting. As indicated in Figure 7D,E, H_2_O_2_ enhanced the conversion of LC3-I to LC3-II and induced Beclin-1 expression, but downregulated the expression of p62. However, these changes caused by H_2_O_2_ were all abrogated by phloroglucinol, supporting flow cytometry results that H_2_O_2_-mediated autophagy could be protected by phloroglucinol.

## 4. Discussion

In the current study, we induced oxidative stress using H_2_O_2_ to examine whether phloroglucinol could protect human RPE ARPE-19 cells from oxidative injury. We found that H_2_O_2_ induced apoptosis, accompanied by mitochondrial dysfunction, DNA damage, and autophagy through an increase in ROS generation. However, phloroglucinol was able to block H_2_O_2_-induced cellular damage and scavenge ROS.

Induction of cytotoxicity including DNA damage and cell death by oxidative stimulation is mostly accompanied by mitochondrial dysfunction associated with ROS generation [33,34]. In healthy retinal cells, ROS levels remain low as a result of normal cellular metabolism. However, accumulation of ROS caused by oxidative stress can act as an initiator in the pathogenesis of degenerative diseases of the retina [4,35]. In this study, inhibition of cell survival, induction of cytotoxicity, and generation of ROS by H_2_O_2_ in ARPE-19 cells were significantly suppressed by pretreatment with phloroglucinol or NAC, a free-radical scavenger used as a positive control. These results showed the possibility that phloroglucinol could block ROS generation caused by oxidative stress. Many previous studies have shown that DNA damage and apoptosis can be induced in RPE cells exposed to oxidative stimuli [35,36]. This finding was also confirmed in H_2_O_2_-treated ARPE-19 cells. We first performed a comet assay, a widely used method to detect DNA strand breaks in eukaryotic cells [37], to evaluate whether pretreatment of phloroglucinol could inhibit H_2_O_2_-induced DNA damage. We found that phloroglucinol effectively inhibited the comet tail moment (DNA migration) observed in cells treated with H_2_O_2_. In addition, the expression of p-γH2AX, a biomarker of DNA double-strand break [38], and the amount of 8-OHdG, an indicator of oxidative stress-mediated DNA damage [39], were increased by H_2_O_2_ treatment. However, these changes were all canceled by treatment with phloroglucinol. The blocking effect of phloroglucinol on these three indicators was similarly observed in cells pretreated with NAC. Our results well support results shown in H_2_O_2_-treated human keratinocytes and UVB-irradiated mouse skin [18,23,24]. These results suggest that the ROS scavenging ability of phloroglucinol might contribute to the reduction in H_2_O_2_-induced DNA damage in RPE cells.

Apoptosis is usually divided into extrinsic and intrinsic pathways. Overload of ROS by oxidative stress can depolarize the mitochondrial membrane, which contributes to the activation of mitochondria-mediated intrinsic apoptosis pathway [40,41], resulting in the collapse of MMP indicative of dysfunctional mitochondria and leading to cytosolic release of cytochrome *c*. Released cytochrome *c* can activate the caspase cascade required for the intrinsic apoptosis pathway, causing degradation of caspase-dependent proteins such as PARP, thereby terminating apoptosis [40,42,43]. As reported in previous studies [23,44,45], the reduction in MMP and cytoplasmic release of cytochrome *c* are major events during mitochondria-mediated apoptosis. These events were increased in H_2_O_2_-treated ARPE-19 cells in the present study. However, there changes were markedly blocked by phloroglucinol. Furthermore, expression of Bcl-2 family proteins, activation of caspase-3, and cleavage of PARP by H_2_O_2_ were maintained at control levels after phloroglucinol pretreatment, in good agreement with our previous study using human keratinocytes [23]. Accumulated prior studies have shown that the intrinsic pathway is critically controlled by Bcl-2 family members. Among them, anti-apoptotic proteins including Bcl-2 are essential to maintain stability of the mitochondrial membrane barrier, whereas anti-apoptotic proteins such as Bax are key executors of mitochondrial poration, thereby enhancing mitochondrial membrane permeability and releasing mitochondrial cytochrome *c* [8,40]. These findings well support our finding that phloroglucinol can prevent apoptosis by suppressing the intrinsic apoptotic pathway. Taken together, our findings indicate that the antioxidant activity of phloroglucinol is responsible for H_2_O_2_-induced blockade of apoptosis in ARPE-19 cells.

Although the primary targets of intracellular ROS are mitochondria, mitochondria are also major sources of ROS. Increased ROS in turn can inhibit mitochondrial efficiency, which can lead to more ROS production in mitochondria by a self-destructive vicious cycle [46,47]. Therefore, we evaluated whether ROS generated by H_2_O_2_ was derived from mitochondria by applying MitoSOX-red, a mitochondrial superoxide indicator, and Mito-TEMPO, a specific antioxidant for mtROS based on previous studies showing that the generation of ROS induced by H_2_O_2_ in ARPE-19 cells occurs in mitochondria [48,49]. MMP lost by H_2_O_2_ was also significantly abolished by treatment with phloroglucinol or Mito-TEMPO alone. However, in cells pretreated with both phloroglucinol and Mito-TEMPO, MMP was almost completely restored to untreated control levels. Moreover, H_2_O_2_-induced cytosolic release of cytochrome *c* and expression of mitophagy markers such as PINK1 and PARKIN were not observed in cells pretreated with phloroglucinol, which might be due to blockade of mtROS production by phloroglucinol. During mitophagy, a type of autophagy unique to mitochondria, PINK1 recruits PARKIN for autophagosome formation, which in turn initiates the removal of damaged mitochondria via autophagy and proteasome mechanisms [50,51]. In AMD-like pathology associated with RPE injury, accumulation of mitochondrial damage and reduction in biogenesis are closely related to the induction of mitophagy, a phenomenon that appears prominently as aging progresses [4,10]. In particular, an aged retina is characterized by increased ROS accumulation, impaired autophagy, and mitochondrial damage associated with the pathogenesis of AMD. Rohrer et al. [52] have demonstrated that RPE cells isolated from eyes of elderly donors are more sensitive to oxidative stress and that a further decrease in mitochondrial metabolism might be associated with increased mitophagy. In addition, Kim et al. [9] have recently shown that mitochondrial dysfunction in H_2_O_2_-injured rat retina and RPE cells is responsible for the induction of mitophagy. As in other cells, oxidative stress-induced mitophagy in RPE cells occurs through the PINK1-PARKIN signaling pathway, a process that clears damaged mitochondria through autophagy [53,54]. Therefore, our results suggest that suppression of mtROS production and preservation of mitochondrial function by phloroglucinol in ARPE-19 cells exposed to H_2_O_2_ are mediated by blockade of mtROS production.

Recently, the importance of autophagy in AMD pathology has been steadily rising. It has been shown that mtROS-mediated autophagy induced by oxidative stress may contribute to retinal damage [55,56]. Autophagy is a critical catabolic process for adapting to metabolic stress and maintaining homeostasis by removing damaged intracellular organelles (including mitochondria) and proteins through formation of autophagosomes. This process is involved in the promotion and inhibition of apoptosis depending on stimulators that induce autophagy, the type of cell, and the environment surrounding the cell [50,51]. One of the features of retinal aging is the accumulation of autophagy proteins associated with mitochondrial damage [57,58]. In this respect, pharmacological manipulation of autophagic activity could be a therapeutic target for retinal damage-related disorders. Although autophagy in RPE cells exposed to oxidative stress, particularly H_2_O_2_, is known to contribute to apoptosis induction [49], RPE cells might also be protected from oxidative stress and apoptosis through promotion of autophagy [59]. In this study, H_2_O_2_-induced autophagy in ARPE-19 cells was blocked by 3-MA, an autophagosome blocker, suggesting that H_2_O_2_-mediated autophagy might contribute to apoptosis induction. Phloroglucinol also conferred a protection against H_2_O_2_-induced autophagy, similar to 3-MA. In addition, as is commonly observed during autophagy, in H_2_O_2_-treated cells, the conversion of LC3-I to LC3-II was increased and p62 was down-regulated while Beclin-1 was up-regulated. However, H_2_O_2_ stimulation in the presence of phloroglucinol failed to induce an increase in LC3-II/LC3-I value or Beclin-1 expression, which could serve as markers of autophagy because they were involved in the formation of autophagosomes [7,60]. On the other hand, p62, an indicator of autophagic flux due to degradation in autolysosomes [61,62], was maintained at the control level. Therefore, phloroglucinol might protect ARPE-19 cells from H_2_O_2_-induced cellular damage, a pro-apoptotic mechanism, by counteracting the process of autophagy.

## 5. Conclusions

**Figure 8:**
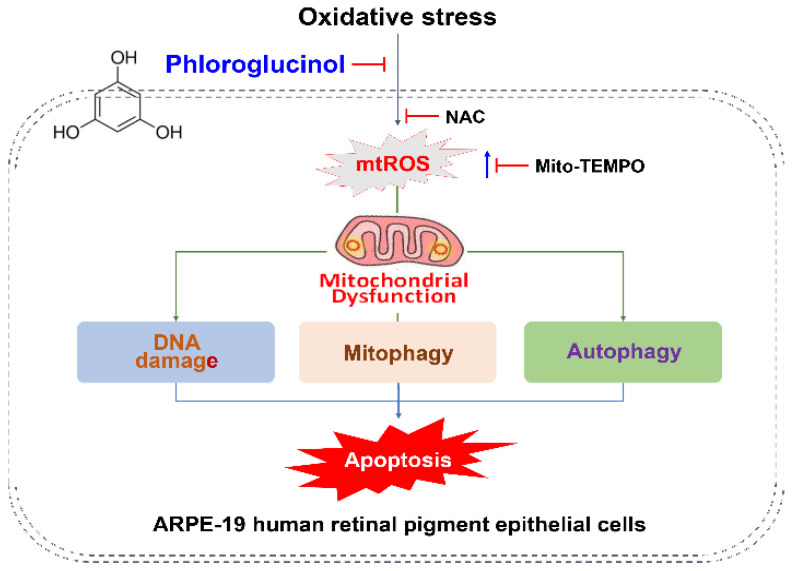
Schematic showing the protective effect of phloroglucinol on oxidative injury in human RPE ARPE-19 cells. As a scavenger of mtROS, phloroglucinol protects against oxidative stress-induced apoptosis by blocking mitochondrial and DNA damage and autophagy.

Taken together, our results suggest that phloroglucinol can protect RPE cells from H_2_O_2_-induced oxidative damage, thereby lessening DNA and mitochondrial damage, reducing apoptosis, and improving cell survival. This protective effect of phloroglucinol can at least be achieved by regulating mitochondrial-mediated autophagy through blockade of mtROS production (Figure 8). Although our results support the beneficial potential of phloroglucinol for the prevention and treatment of oxidative damage-mediated retinal diseases, many limitations need to be addressed. First, further studies on the role of other signaling pathways including intracellular antioxidant signaling and PI3K/AKT/mTOR signaling are needed to clearly understand the blocking mechanism of mtROS by phloroglucinol. Additionally, since only in vitro experiments challenged with H_2_O_2_ were employed, animal experiments should be performed in the future to verify our findings in vivo along with additional studies with other oxidative stress inducers.

## Figures and Tables

**Table 1:** List of antibodies used in this study.

Antibody	Species	Dilution	Catalog No.	Vendor
Bax	Mouse monoclonal	1:1000	sc-7480	Santa Cruz Biotechnology Inc.
Bcl-2	Mouse monoclonal	1:1000	sc-509	Santa Cruz Biotechnology Inc.
Caspase-3	Mouse monoclonal	1:1000	sc-56052	Santa Cruz Biotechnology Inc.
PARP	Mouse monoclonal	1:1000	sc-8007	Santa Cruz Biotechnology Inc.
Pink1	Mouse monoclonal	1:1000	sc-517353	Santa Cruz Biotechnology Inc.
Parkin	Mouse monoclonal	1:1000	sc-32282	Santa Cruz Biotechnology Inc.
Cytochrome *c*	Mouse monoclonal	1:1000	sc-13560	Santa Cruz Biotechnology Inc.
LC3	Rabbit polyclonal	1:1000	3868s	Cell Signaling Technology Inc.
Beclin-1	Rabbit polyclonal	1:1000	3495s	Cell Signaling Technology Inc.
p62	Rabbit polyclonal	1:1000	5114	Cell Signaling Technology Inc.
COX IV	Rabbit polyclonal	1:1000	4844	Cell Signaling Technology Inc.
Actin	Mouse monoclonal	1:1000	sc-47778	Santa Cruz Biotechnology Inc.

## Data Availability

The data are contained within this article.
